# Effect of Welding Current on Weld Formation, Microstructure, and Mechanical Properties in Resistance Spot Welding of CR590T/340Y Galvanized Dual Phase Steel

**DOI:** 10.3390/ma11112310

**Published:** 2018-11-17

**Authors:** Xinge Zhang, Fubin Yao, Zhenan Ren, Haiyan Yu

**Affiliations:** 1 School of Mechanical and Aerospace Engineering, Jilin University, Changchun 130025, China; zhangxinge@jlu.edu.cn; 2 College of Materials Science and Engineering, Jilin University, Changchun 130025, China; 3 Doosan Infracore China Co., Ltd., Yantai 264006, China; yaofbjlu@126.com; 4 Sanyou Automobile Parts Manufacturing Co., Ltd., Changchun 130022, China; yuhy_sy@163.com

**Keywords:** Resistance spot welding, galvanized dual phase steel, microstructure, mechanical properties, weld formation

## Abstract

During resistance spot welding, the welding current is the most important process parameter, which determines the welding heat input and then has a great influence on the welding quality. In present study, the CR590T/340YDP galvanized dual phase steel widely used as automobile material was carried out using resistance spot welding. The effect of welding current on the weld formation, microstructure, and mechanical properties was studied in detail. It was found that the quality of weld appearance decreased with the increase of welding current, and there was a Zn island on the weld surface. The microstructure of the whole resistance spot welded joint was inhomogeneity. The nugget zone consisted of coarse lath martensite and a little of ferrite with the columnar crystal morphology, and the microstructure of weld nugget became coarser when the welding current was higher. There was an optimum welding current value and the tensile strength reached the maximum. This investigation will provide the process guidance for automobile body production.

## 1. Introduction

In the automotive field, reducing the body weight is one of the most fundamental ways to save energy and reduce pollution. Therefore, more and more lightweight and high-strength materials have been developed for body manufacturing [1,2,3]. Galvanized dual phase steel has the advantages of low yield ratio, good formability, high tensile strength, good matching of strength and plasticity, corrosion resistance, and so on, which has been used in automobile body manufacturing [4,5,6]. The wide application potential of galvanized dual phase steel in the automotive field mainly depends on the welding methods and its welding quality.

In previous literature, several welding methods, such as laser welding [7,8,9,10], gas metal arc welding (GMAW) [11,12,13], and friction stir welding (FSW) [14,15,16], have been employed to weld the galvanized dual phase steel, and these welding methods have their own advantages and disadvantages, as well as being applicable to different base metal materials and joint shapes. Resistance spot welding has the advantages of high efficiency and low cost, which is an important welding method for manufacturing the automobile sheet structures [17,18]. Many scholars have studied the microstructure, softening zone characteristics, mechanical behavior, and welding spatter defect of resistance spot welding of dual phase steels [19,20,21,22,23]. The CR590T/340YDP galvanized dual phase steel sheet used as an automobile manufacturing material not only has good corrosion resistance, but also high strength, which can effectively reduce the weight of the car body, and some research on resistance spot welding have been carried out [24,25,26]. Wang et al. [27] investigated the effect of base material chemical compositions on the properties of the resistance spot welding joint of DP590 steel, and the results indicated the tensile strength and toughness of welded joints were affected by the chemical compositions of the base material, especially the carbon content. Namely, for the same grade DP590 steel, the weld formation, microstructure, and mechanical properties of resistance spot welding will be different if the chemical compositions of the base material are not the same. During the resistance spot welding process, it is well known that the welding heat generation can be expressed as Q = *I*^2^*Rt* (*I* is the welding current; *R* is the resistance; and *t* is the welding time). In general, the welding time and welding current are 10^−1^ *s* level and kA level, respectively. Therefore, the welding current is considered as the key factor to determine the welding heat input and influence of the welding quality [28]. Wang et al. [29] established a finite element model for resistance spot welding of DP590 steel, and the nugget formation process was investigated. The simulative result for nugget size was obviously bigger than that of the experiment under a large welding current, although they were well fitted under a suitable welding current. Therefore, the experimental investigation on the effect of the welding current on properties in resistance spot welding of DP590 steel has an important significance.

To explore the weldability, and provide the process with guidance for automobile body production, the CR590/340Y galvanized dual phase steel sheets employed for the automobile front longitudinal beam part were carried out by resistance spot welding, and the influence of the welding current on weld formation, microstructure, microhardness, and tensile strength was studied in detail.

## 2. Materials and Methods

**Figure 1:**
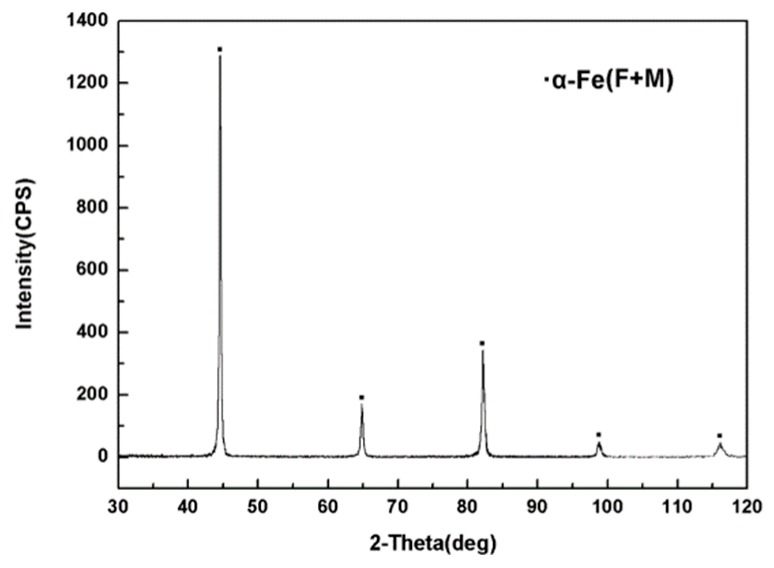
XRD pattern of base metal.

In this study, the base metal is galvanized dual phase steel (Trademark: CR590T/340Y) sheet, which is always used to manufacture the automobile’s front longitudinal beam part. The thickness of the base metal is 2 mm, and its .hemical compositions are listed in Table 1. The XRD pattern result indicates that the base metal consisted of ferrite (86.2 vol.%) and martensite (13.8 vol.%), as shown in Figure 1. The galvanized dual phase steel sheets were conducted with double-side hot galvanizing and the weight of the Zn layer is 80 g/m^3^. The yield strength and tensile strength of the base metal are 356 MPa and 605 MPa, respectively.

**Figure 2:**
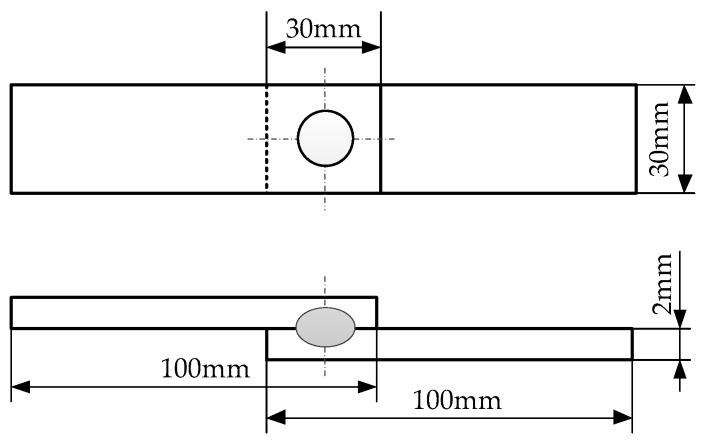
Schematic diagram of dimensions and configuration of resistance spot welding joint.

The resistance spot welding robot (Shougang MOTOMAN Robot Co., Ltd., Beijing, China) was employed to weld the overlap joints, and the working face diameter of Cu-Cr-Zr alloy electrodes was 6 mm. The dimensions and configuration of the joint are indicated in Figure 2. The welding time and electrode force were set at 20 cycles and 4.0 kN by preliminary process experiments, and the main experimental parameters are given in Table 2.

After welding, the appearance of the resistance spot weld was observed by digital camera (Canon Ixus1000, Cannon, Tokyo, Japan). The resistance spot weld was cut from weld nugget center, and then mounted, polished, and etched for the microstructure observed and analysis. The etching solution was 4 vol.% nitric acid. The microstructure of base metal, heat affected zone, and weld nugget was examined by Evol-18 scanning electron microscopy (SEM) (Carl Zeiss, Jena, Germany) with energy dispersive spectroscope (EDS). The phase structures were identified using the X-ray diffractometer (XRD) (D/MAX-2500PC, Rigaku, Tokyo, Japan) machine with 50kV voltage, 300 mA current, Cu Kα radiation, and 4 °/min scanning rate. The Vickers hardness was measured on the cross section of the welded joint using an MH-3 microhardness test machine (Shanghai Tuming, Shanghai, China), and the test load and load time were 1.961 N and 10 s, respectively. The tensile shear test was performed using CSS-44100 material testing equipment with a 100 kN maximum load (China Mechanical Testing Equipment Co., Ltd., Changchun, China), and the strain speed was 6 mm/min.

## 3. Results and Discussion

### 3.1. Weld Formation

#### 3.1.1. Weld Appearance

**Figure 3:**
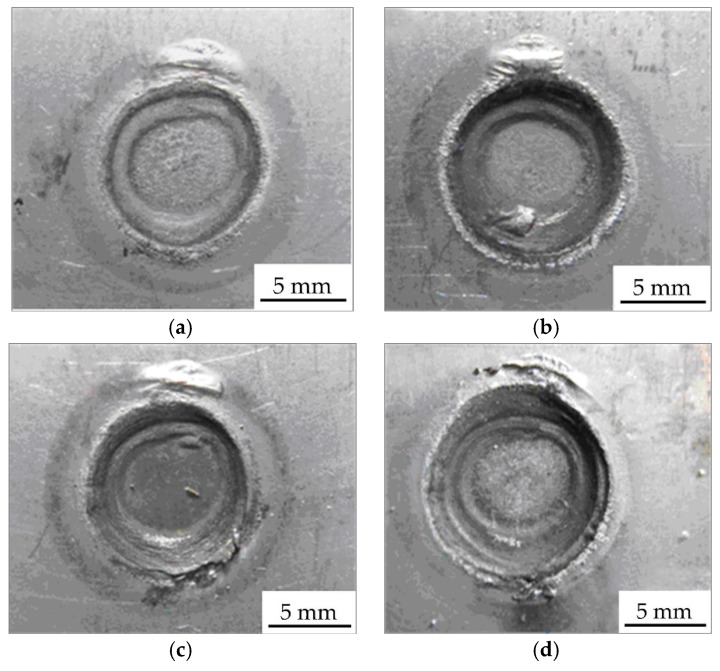
Resistance spot weld appearance gained with different welding currents: (**a**) 8.5 kA; (**b**) 10.0 kA; (**c**) 11.0 kA; (**d**) 12.0 kA.

The resistance spot weld appearance depends on the coupling effect of heat, electric, and load, which will influence the welded joint quality, corrosion resistance, and look. Figure 3 shows the appearances of typical resistance spot welded joints, which were gained with different welding currents. During the resistance spot welding process, while the welding current was 8.5 kA~10.5 kA, there was no welding spatter on the spot weld surface. Until the welding current was 11 kA, the welding spatters were generated, and the welding spatters obviously increased. The welding spatter is caused by the large welding heat input, which results in the faster speed of melting metal than the expansion speed of the plastic ring, and the melted liquid metals fly out of the plastic ring. Because the welding current is the main parameter to determine the heat input (*Q* = *i*^2^*Rt*, *Q* is the welding heat input, *i* is the welding current, and *t* the is welding time), the welding heat input increases sharply when the welding current is increased, then the high heat input brings about more welding spatters.

**Figure 4:**
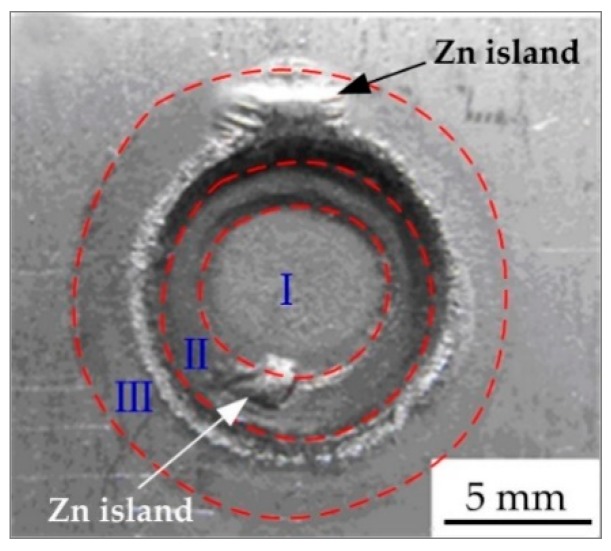
Three feature zones on the resistance spot weld surface.

**Figure 5:**
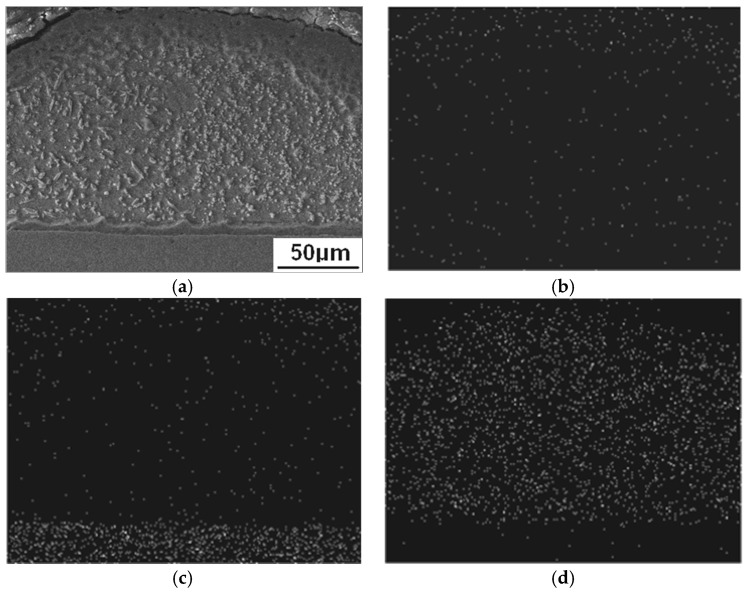
SEM image and element map distribution of Zn island: (**a**) SEM image; (**b**) O Kα 1; (**c**) Fe Kα 1; (**d**) Zn Kα 1.

Figure 4 indicates the three feature zones on the resistance spot weld surface, and it comprises of three circle zones (circle zone I, circle zone II, and circle zone III) from the center to the outside. The circle zone I was the area where the electrodes contacted with the base metal during the resistance spot welding process, and is located in the centre of the spot weld surface. It was produced by various physical factors, such as the heat, electricity, and load, of the electrodes and the binding force of the base metal. Because of the highest temperature in the circle zone I and the low melting point of Zn (692 K), the welding heat caused the Zn layer to melt and be squeezed away by the electrodes, meanwhile the base metal substrate was exposed and oxidized. The circle zone II is located outside the adjacent region of the circle zone I. The profile of the circle zone II depended on the working surface shape of the electrodes and the indentation depth of the spot welded joint. On the circle zone II, the Zn extruded from circle zone I along the edge of the electrodes, and molten Zn on circle zone II was aggregated by the action of gravity and surface tension, then solidified to form the Zn island as shown in Figure 4. The SEM image and element (white spots in Figure 5b–d) map distribution of the Zn island (on the circle zone II in Figure 4 as an example) are shown in Figure 5, and the main components of the Zn island comprised of O (8.57%), Fe (15.44%), and Zn (65.24%). The circle zone III was the heat affected zone of spot welded joints. The base metal in this zone was heated and the Zn layer was oxidized to form ZnO. The Zn island also could generate on the circle zone III.

**Figure 6:**
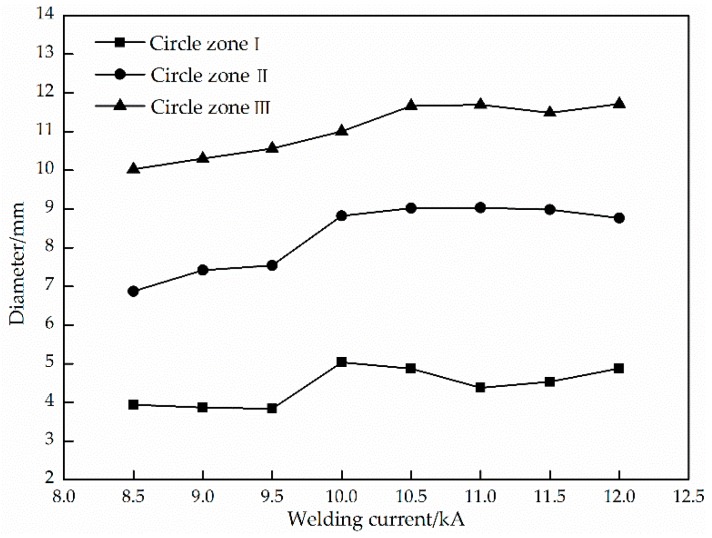
Effect of the welding current on sizes of three feature zones on the weld appearance.

From Figure 3a, due to the low welding current, the Zn on the surface of circle zone I was partially melted and the color was not much different from that on the base metal; the profile of circle zone II was small with a smooth transition, and the Zn island was generated in the circle zone III. While the welding current was 10.0 kA, the Zn layer on the surface of the circle zone was not seriously damaged, and the inner and outer colors of circle zone II were quite different. The Zn island was formed on the adjacent part of circle zone I, because the Zn layer on the circle zone II was melted and extruded, and then cooled and crystallized on the outside. There was a Zn island on the circle zone III. When the welding current continued to increase to 11.0 kA, the Zn layer on the circle zone I was seriously damaged. There was a Cu-Zn alloy formed by the reaction of the molten Zn layer and copper on the edge of circle zone II, and the electrodes’ adhesion occurred. When the welding current reached 12.0 kA, the appearance quality of the spot welded joint decreased obviously and many spatters were generated because of the uneven heat distribution of the electrodes. Figure 6 indicates the relation between the welding current and the diameters of the three feature zones on the weld appearance. When the welding current was 8.5~9.5 kA, the diameters of circle zone I were almost unchanged. With the continued increase of the welding current, the diameters of circle zone I increased to the maximum at first and then decreased gradually. The diameter of the circle zone II changed little with a low welding current, which was the largest with a 10.5 kA current. The diameter of the circle zone III increased with the increase of the current from 8.5 kA to 10.5 kA, but when the welding current was higher than 10.5 kA, the diameter of circle zone III did not change obviously. The results displayed that the welding current had an important influence on the weld appearance, and a low welding current was used on the basis of meeting the strength requirements of the welded joint.

#### 3.1.2. Main Dimensions of Welded Joint Cross-Section

**Figure 7:**
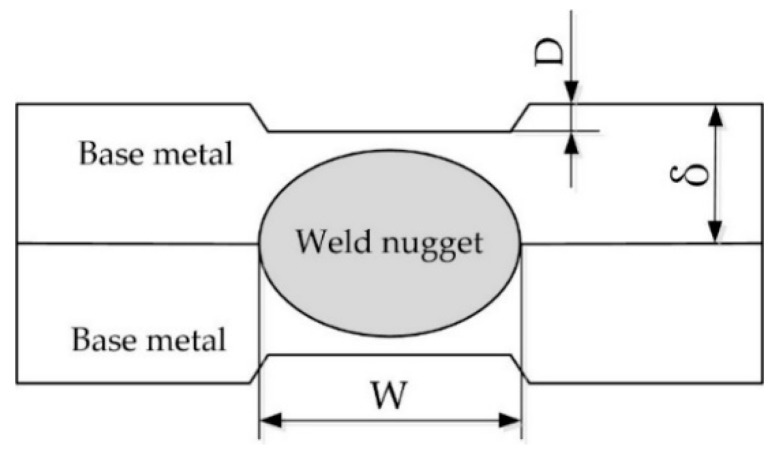
Schematic diagram of the main dimensions of the welded joint cross-section.

The main dimensions of the resistance spot welded joint cross-section include the indentation (usually expressed by indentation rate, D/δ) and weld nugget width at the overlap surface (W), as shown in Figure 7. The indentation influences the weld appearance smoothness, reduces the welded joint cross-section size, and causes stress concentration, which results in reducing the strength of the welded joint. The tensile strength of the resistance spot welded joint is mainly controlled by the W.

**Figure 8:**
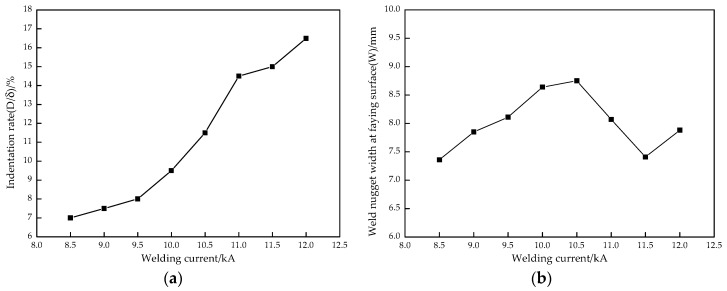
Relationships between the welding current and (**a**) indentation rate (D/δ); (**b**) weld nugget width at overlap surface (W).

The effect of the welding current on the indentation rate is indicated in Figure 8a. The results indicated that the indentation rate was small and increased less with a low welding current because the welding heat input was small, which caused a small amount of base metal melting. When the welding current was between 9.5 kA and 11.0 kA, the welding heat input increased rapidly, so more base metal was melted and the indentation rate increased. If the welding current was greater than 11.0 kA, the indentation rate increased gradually due to welding spatters and other defects, and the indentation was too serious to satisfy the welding quality requirements. Figure 8b displays the relationship between the welding current and the W. The weld nugget width increased rapidly from 7.36 mm to 8.64 mm with an increase of the current from 8.5 kA to 10.0 kA, and the maximum value was 8.75 mm at the 10.5 kA welding current. While the current changed from 10.0 kA to 10.5 kA, the welding heat input reached a quasi-steady state and the change of the weld nugget width was small. When the welding current was greater than 10.5 kA, the current density was higher, and a large number of welding spatters were generated, which reduced the amount of melted base metal in the weld nugget and thus decreased the weld nugget width. If the welding current continued to increase to 12.0 kA, the weld nugget width increased because of the larger welding heat input, but there were many welding spatters, and also some shrinkage and crack defects in the weld nugget.

### 3.2. Microstructure

**Figure 9:**
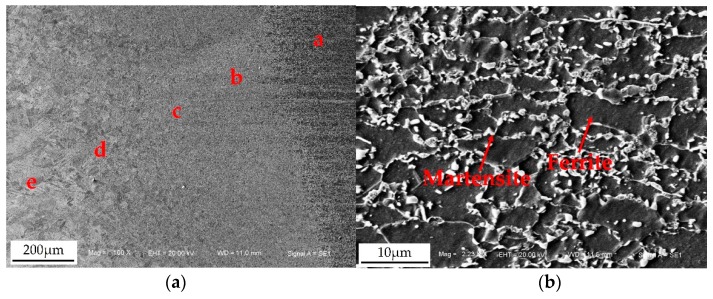

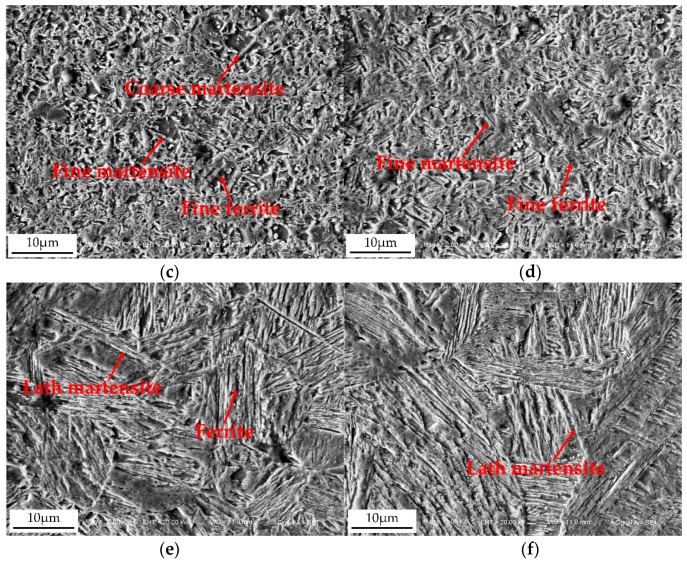
Microstructure of (**a**) different zones of the welded joint; (**b**) base metal; (**c**) uneven structure zone; (**d**) fine grain zone; (**e**) superheated zone; (**f**) weld nugget zone.

Due to the uneven heat input and different cooling conditions, the microstructure of the resistance welded joints was very inhomogeneous. As shown in Figure 9a, the whole resistance spot welded joint comprised of the base metal (a zone), uneven structure zone (b zone), fine grain zone (c zone), superheated zone (d zone), and weld nugget zone (e zone). The microstructure of the base metal consists of ferrite and martensite as shown in Figure 9b. The grain and microstructure in the uneven structure zone were obviously heterogeneous, as shown in Figure 9c. Because the temperature in this zone was between Ac_1_ and Ac_3_ during the resistance spot welding process, the phase transformation and recrystallization occurred for part of the base metal, and the fine ferrite and martensite formed; meanwhile, the ferrite, which was not austenitized, became the coarse ferrite. Therefore, there were also similarities to the structure of the base metal in this zone. During the welding process, the base metal in the fine grain zone was heated to above Ac_3_, and all ferrite and martensite were recrystallized to austenite. The fine and homogeneous ferrite and martensite were obtained after cooling, which were similar to the normalized microstructure of heat treatment, as shown in Figure 9d. The overheated zone consisted of coarse lath martensite and a little of the ferrite shown in Figure 9e. The temperature in this zone was between 1100° and the solidus temperature, and the austenite was overheated. The grain grew up seriously and the chemical compositions in the grain were uniform, hence, the coarse martensite was obtained after rapid cooling. The nugget zone comprised of coarse lath martensite and a little of ferrite with the columnar crystal morphology, as shown in Figure 9f. Because the maximum temperature gradient in the nugget zone was along the axis of the electrodes, the molten liquid metal nucleated and crystallized at the fusion line first, and then formed the columnar austenite along the direction of the higher temperature gradient. Finally, the solid austenite transformed into martensite because of the rapid cooling rate of the welding process and low carbon component in the base metal.

**Figure 10:**
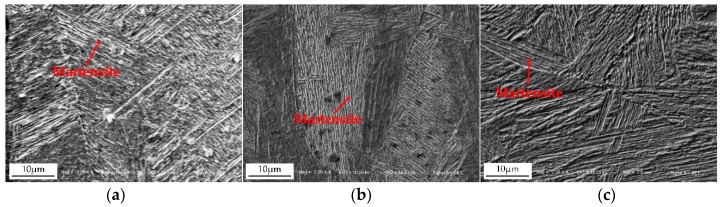
Effect of the typical welding current on the microstructure of the weld nugget: (**a**) 8.5 kA; (**b**) 10.5 kA; (**c**) 12 kA.

The weld nugget is the important zone of resistance spot welded joints, and its microstructure characteristics directly affected the mechanical properties of welded joints. The heat generation and heat transfer in the nugget zone were different under different welding currents, so the microstructure was very different. Figure 10 shows the influence of a typical current on the microstructure of the weld nugget. It can be seen that while the welding current was low, the welding heat input was low, and the weld nugget was mainly lath martensite with a fine and uniform structure. At the same time, the plastic deformation of the weld nugget zone was large, and there was no welding defect in the weld nugget zone. While the welding current was 10.5 kA, the welding heat input increased and the microstructure became coarser. The decrease of the cooling rate resulted in the decrease of martensite and the increase of ferrite. The microstructure of the weld nugget was relatively uniform, and there was no obvious welding defect. If the welding current was the maximum value of 12.0 kA, a large number of spatters caused some heat loss and a rapid cooling rate, so that the weld nugget microstructure consisted of lath martensite. The grains in the weld nugget center grew up and coarsened greatly, as shown in Figure 10c. In short, when the welding current varied from 8.5 kA to 12 kA, the weld nugget columnar structure gradually coarsened, mainly because of the larger heat input and the reduction of the cooling rate.

### 3.3. Microhardness Distribution

**Figure 11:**
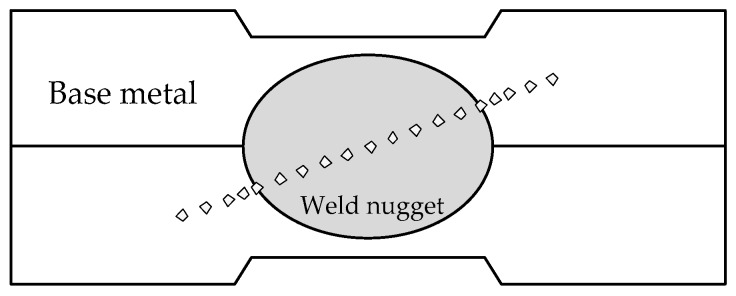
Schematic diagram of the microhardness measurement location of the welded joint.

The microhardness measurement was performed on the cross section of the resistance spot welded joint. The schematic diagram of the measurement location is shown in Figure 11. The distance between two test points in the base metal and weld nugget zone was 0.5 mm. Because the HAZ width was narrow, the two test points distance was 0.25 mm in HAZ.

**Figure 12:**
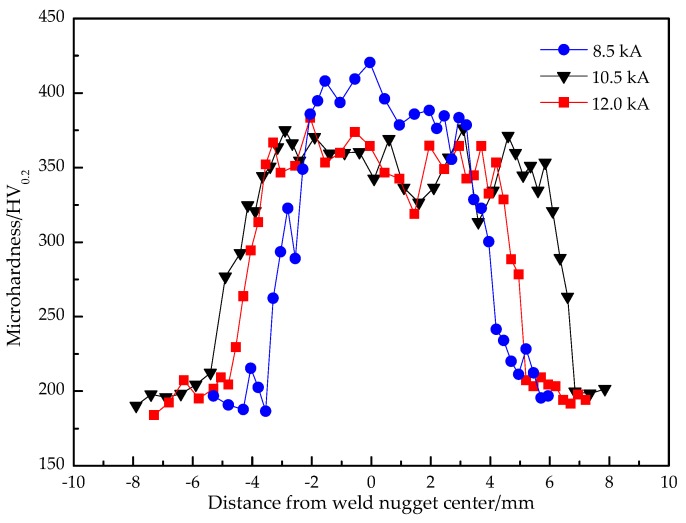
Microhardness distributions of welded joints with different welding currents.

Figure 12 displays the microhardness distributions of resistance spot welded joints gained with different welding currents. The microhardness of the base metal was 198 HV_0.2_, and the microhardness in HAZ was obviously higher than the base metal. The microhardness of the weld nugget zone was the highest, which was above 350 HV_0.2_. The microhardness at the edge of the weld nugget was slightly higher than that of the weld nugget center. Due to the uneven distribution of the current density at the welding joint, the current density at the edge of the weld nugget was greater than the average current density, which generated a greater resistance heat. Therefore, during the resistance spot welding process, the solidification and crystallization first occurred at the edge of the weld nugget. Meanwhile, the temperature gradient was large and the cooling rate was fast, so the martensite was large and coarse and the microhardness was higher in this zone. With the increase of the welding current, the average microhardness in the weld nugget zone decreased. While the welding current was 8.5 kA, the microhardness of the weld nugget was higher because of the faster cooling rate, and the microstructure was lath martensite, which was uniform and fine, so the hardness was higher. While the welding current was 10.5 kA, the cooling rate should decrease. The columnar crystals of the weld nugget grew up, mainly composed of lath martensite and acicular ferrite, which resulted in the decrease of hardness. When the welding current continued to increase to 12.0 kA, the microhardness of most zones of the weld nugget was higher than that of 10.5 kA due to the large heat input and coarse structure, but there were welding defects in the center of the weld nugget, so that the average value of microhardness decreased.

### 3.4. Tensile Shear Strength

**Figure 13:**
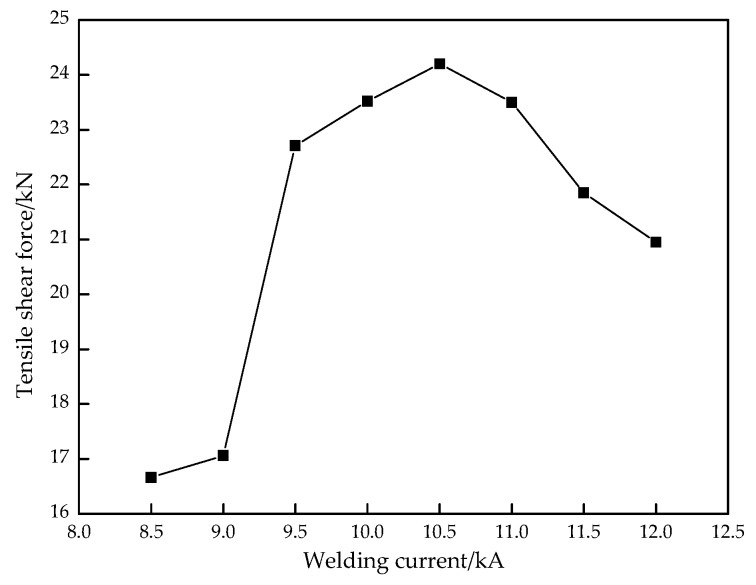
Effect of the welding current on the tensile shear force of welded joint.

The tensile shear force is often used to characterize the welded joint strength. The larger the tensile shear force is, the better the strength is. Figure 13 indicates the effect of the welding current on tensile shear force. Compared with Figure 8b, it can be found that the variation trend of the W and tensile shear force with the welding current is basically the same; that is, the larger the weld nugget width, the greater the tensile shear force. With the first increase of welding current, the weld nugget width and tensile shear force all obviously increased. While it was 10.5 kA, the weld nugget width and tensile shear force all reached the maximum value of 8.75 mm and 24.20 kN, respectively. Subsequently, the weld nugget width dropped rapidly and an inflection point appeared while the welding current was 11.5 kA, but the tensile force decreased continuously. With the large welding current, the melting amount of the base metal increased gradually, which caused the increase of the weld nugget width. Thus, the bonding strength of the resistance spot welded joint increased and the tensile shear force increased. However, when the welding current was 12.0 kA, the welding heat input was too large, resulting in a large number of spatters, shrinkage cracks, and other defects in the welded joint. Although the weld nugget width increased, the effective bonding width of the welded joint decreased, so the tensile shear force decreased.

**Figure 14:**
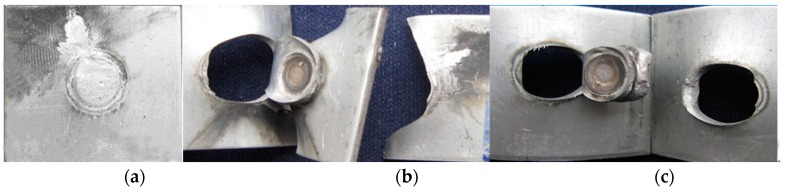
Three failure modes of the resistance spot welded joints: (**a**) Interface failure; (**b**) pullout failure (base metal tear fracture); (**c**) pullout failure (pullout tear fracture).

**Figure 15:**
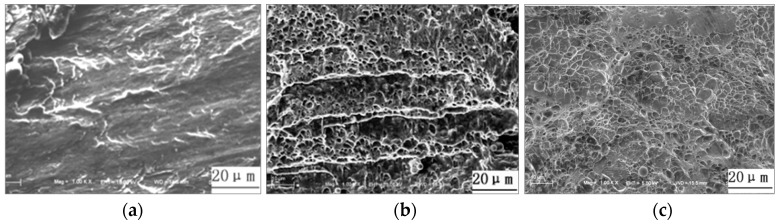
SEM images of the fracture surface: (**a**) Interface failure; (**b**) pullout failure (base metal tear fracture); (**c**) pullout failure (pullout tear fracture).

During the tensile shear experiment, there were two failure modes: Interface failure and pullout failure, as shown in Figure 14. The SEM images of fracture surfaces were indicated in Figure 15. The welded joints, which were gained with the lower welding current (≤9.5 kA) and higher welding current (≥11 kA), always ruptured along the overlap surface, as shown in Figure 14a. While the welding current was low (≤9.5 kA), the W was narrow, as well as the strength of the base metal was high. Therefore, under the tensile shear force, the crack produced on the weld nugget edge at the overlap surface at first and then extended along the overlap surface until the welded joint failed with the interface failure mode. While the welding current was high (≥11 kA), the bonding strength of welded joints was small due to the welding defects, such as spatter, shrinkage, and cracks, in the weld nugget, which resulted in the smaller tensile shear force for the welded joint, and its tensile shear specimen also ruptured along the overlap surface. As shown in Figure 15a, the river pattern was obvious on the fracture surface, which illustrated the brittle fracture characteristics. While the welding current was 10 kA, the fracture pattern of the welded joint was the pullout failure mode, as shown in Figure 14b. It can be found that the fracture surfaces are mainly dimples and a small amount of cleavage steps from Figure 15b. Under the tensile shear force, the tensile specimen first produced necking in the HAZ of the welded joint. With the increase of tensile shear force, the dimples grew up and converged, and then the tensile specimen broke down in the base metal. In the present study, the weld nugget width and tensile shear force were all maximum with a 10.5 kA welding current. During the tensile shear test for welded joints produced with a 10.5 kA welding current, because of the large weld nugget and few welding defects, the welded joint was not easy to rupture from the overlap surface. The tensile stress on the edge of the weld nugget increased gradually, owing to the angle between the overlap surface and tensile force. The HAZ was the weakest zone due to the inhomogeneous microstructure, coarse grains, and low plasticity and toughness. With the increase of tensile force, the necking occurred first in the HAZ, and a number of micro-holes began to form in the center of the necking. The micro-holes grew and formed the dimples, which converged to form the crack. Finally, the crack was torn along the HAZ to form the pullout tear failure, as show in Figure 14c. Figure 15c shows the pullout tear fracture surface, which was mainly composed of small and uniform dimples.

## 4. Conclusions

(1) The Zn island was generated on the resistance spot weld surface because of the melting and aggregating of the Zn layer under the action of heated and squeezed by the electrodes. While the welding current increased from 8.5 kA to 12.0 kA, the indentation rate kept growing to 16.5% due to the increase of the welding heat input. However, the weld nugget width obviously increased at first, which reached the maximum when the welding current was 10.5 kA, and then decreased.
(2) The whole resistance spot welded joint comprises of the base metal, uneven structure zone, fine grain zone, superheated zone, and weld nugget zone. The nugget zone was mainly comprised coarse lath martensite and little ferrite with columnar crystal morphology due to the high temperature gradient and rapid cooling rate. With the increase of the welding current, the microstructure of the weld nugget became coarser; meanwhile, the martensite decreased and the ferrite increased.
(3) The microhardness of the weld nugget zone was highest and the base metal was lowest. With the increase of the welding current, the average microhardness in the weld nugget zone decreased. While the welding current increased from 8.5 kA to 10.5 kA, the tensile shear force was obviously raised, owing to the increase of the weld nugget width. The tensile shear force reached the maximum value of 24.20 kN with a 10.5 kA welding current. If the welding current increased continuously, the tensile shear force decreased because of a large number of spatters and the high indentation rate. The failure modes mainly depended on the weld nugget width at the overlap surface and welding defect. Therefore, while the CR590T/340YDP galvanized dual phase steel sheets with 2 mm thickness were performed using resistance spot welding, the recommended welding current was 10.0~10.5 kA with a 20 cycles welding time and 4.0 kN electrode force.

## Figures and Tables

**Table 1:** Chemical compositions of base metal (wt.%).

Component	C	Si	Mn	S	P	Al	Cr	Nb	Mo	Fe
wt%	0.087	0.008	1.694	0.007	0.001	0.030	0.160	0.012	0.011	Balance

**Table 2:** Experimental parameters.

Parameter	Welding Current (kA)	Welding Time (Cycle, 1 Cycle = 0.02 s)	Electrode Force (kN)
Value	8.5~12.0	20	4.0
